# Development and Validation of an Interprofessional Community-Based Teaching-Learning Module for a Family Adoption Programme for Indian Medical Undergraduates

**DOI:** 10.7759/cureus.67150

**Published:** 2024-08-18

**Authors:** Praveen Ganganahalli, Animesh Jain, Amol Dongre, Rekha Udgiri

**Affiliations:** 1 Community Medicine, Bijapur Liberal District Educational (BLDE) University (Deemed to be University) Shri B M Patil Medical College Hospital and Research Centre, Vijayapura, IND; 2 Community Medicine, Kasturba Medical College, Mangalore, IND; 3 Community Medicine, Manipal Academy of Higher Education, Manipal, IND; 4 Community Medicine, All India Institute of Medical Sciences, Nagpur, IND

**Keywords:** relevant, content validity, community, family adoption, module

## Abstract

Introduction: Medical education aims to prepare graduates to address society’s health requirements effectively. The majority of assessments are summative and provide a minimal chance for feedback. The evaluation techniques and teaching-learning activities place a greater emphasis on knowledge than on attitude and abilities. They might also lack soft skills, including professionalism, ethics, doctor-patient relationships, and communication, as they are a hidden part of the traditional curriculum. Learning activities that take place in a specific setting, like a community, are referred to as community-based education.

Objectives: To develop and validate the Interprofessional Teaching-Learning Module for the Family Adoption Program for medical undergraduates.

Method: This was an educational observational study to develop a module for a family adoption program for Bachelor of Medicine and Bachelor of Surgery (MBBS) students, which began from the first professional year and extended to the second and third professional years. Experts from different health professions were identified, and a preliminary meeting was called to discuss the purpose of the module and the role and responsibilities of each. Institutional Ethics Committee clearance was obtained after the submission of the synopsis.

Results: An interprofessional team was formed and communicated regarding the purpose of the module, and the initial draft was prepared with their contribution. The panel of experts for relevancy validated the module, and the content validity index derived, which was 0.92, was considered good and relevant.

Conclusion: The Interprofessional Teaching-Learning Module for the Family Adoption Program is a comprehensive and collaborative approach for equipping students’ necessary knowledge and skills to facilitate successful adoption and support families effectively.

## Introduction

Medical education aims to prepare graduates to address society’s health requirements effectively. Most assessments are summative and provide a minimal chance for feedback [1]. The teaching-learning activities and the assessment methods focus more on knowledge than attitude and skills [2]. Students might also lack soft skills, including professionalism, ethics, and doctor-patient relationships, as they were a hidden part of the traditional curriculum. Community-based education refers to learning activities in the community setting [3,4]. Community-based education is now recognized as an essential addition because graduates need skills in the community more than in tertiary hospitals [3].

Through community participation, medical students can witness firsthand the living circumstances of the individuals they treat as hospital patients. Additionally, the students comprehend how numerous health variables affect patients in the actual world. As of 2022, Bachelor of Medicine and Bachelor of Surgery (MBBS) students are required to participate in the Family Adoption Programme (FAP) by the National Medical Commission (NMC) [5]. The FAP aims to provide an experiential learning opportunity to Indian medical students towards community healthcare with objectives of giving orientation to primary healthcare, creating health-related awareness within the community, functioning as a first point of contact for any health issue, acting as a conduit between the population and relevant healthcare and generate and analyse data for improving health outcome and evidence-based clinical practice [6]. There should be a minimum of three (five desirable) families assigned to each MBBS student. The student must build rapport, comprehend their health and related factors, and contribute to bettering the family’s and, consequently, the community’s healthcare. It should, therefore, contribute to realising universal health coverage [7].

Interprofessional education (IPE) has been identified as a valuable method of learning experiences that can increase the collaboration and communication of health professionals in healthcare settings. Several studies have reported positive student perceptions of IPE, including improved patient and community outcomes [8].

Even though the NMC has given the guidelines for the FAP but it is the responsibility of the faculty and nodal department to implement it effectively and fulfil the objectives. With this background, a study was planned to develop and validate an Interprofessional Community-Based Teaching-Learning Module for a Family Adoption Program for medical undergraduates.

## Materials and methods

An observational study was planned to develop and validate an interprofessional teaching-learning module for undergraduate students posted in the Community Medicine Department for the FAP is a compulsory part of the Competency Based Medical Education (CBME) curriculum for specified hours (total of 78 hours) as per NMC guidelines, which runs from the first professional year (27 hours) and extend to second year (30 hours) and is to be completed in a third professional year (21 hours). 

Experts from different professions were identified (Medical - medicine, obstetrics and gynecology, pediatrics, pathology, community medicine; nutritionist, bio-statistician, public health nurse), and a preliminary meeting was called to discuss the purpose of the module and the role and responsibilities of each. The module preparation work was started after obtaining consent from all the members and ethics approval from the Institutional Ethics Committee of Bijapur Liberal District Educational (BLDE) University (Deemed to be University), Vijayapura in March 2023 (approval BLDE(DU)/IEC/ 847/2022-23). 

The initial draft of the module is prepared based on the guidelines given by the NMC [6] and sent to the experts for suggestions, scoring and comments. The contents of the module consisted of background, objectives of the module and FAP, timetable for FAP for each year, contents to be covered during the sessions, teaching-learning-assessment methods, feedback form and proforma for family information collection. The primary contents of the module covered are shown in Table 1, which consists of activities to be conducted during the sessions and various teaching-learning-assessment methods used during the FAP in the field and the classroom sessions. 

**Table 1:** Major contents covered in the teaching-learning module FAP - Family Adoption Program NSS - National Service Scheme OSPE - Objective Structured Practical Examination DOAP – Demonstration Observation Assist Perform NMC - National Medical Council

Activities to be conducted	Orientation to FAP & allotments of families	Initial orientation session followed by allotment of three (desirable 5) families in the field by the Community Medicine department
	Collection of socio-demographic-clinical information	By using standardized proforma developed based on the sample proforma provided by NMC
	Anthropometric & systemic examination	The standard procedure of examination is followed (Nutritionist & Community Medicine)
	Community clinic or camp, investigations & treatment	Multidiscipline and interprofessional teams will conduct community camps (Pathology, Medicine, Pediatrics & Obstetrics and gynaecology)
	Follow-up	Follow-up of families after camp (Medico-social worker, Public health nurse. Community Medicine)
	Environment protection activities	In collaboration with NSS, activities like plantation drives, cleaning drives, etc, will be organized by Community Medicine department
	Health education sessions	Medico-Social Worker & Public health nurse will facilitate the program (Community Medicine)
	Feedback to the family	data analysis (Biostatistcian) Feedback to the family is given during the last visit each year by the students under the supervision of the Community Medicine department.
Teaching-learning methods	Orientation lecture	Classroom interactive
	Small group discussion	Small batches of 25 - 30 students
	DOAP – Demonstration Observation Assist Perform	Demonstration in the classroom and performance in the community
	Family case presentation	Community case presentation
Assessment methods	OSPE (Objective Structured Practical Examination)	Based on the skills or competency
	Checklist for family case presentation	Based on a standardized checklist
	Assessment of logbook completion	Based on a standardized checklist
	Environment protection and health education activities	With NSS plantation drive, cleaning activity, health education
	Reflection & Feedback	Based on what was done and what next

Validating the teaching-learning module was conducted by a panel of experts from the field of subject and health profession education. Ten experts were identified and sent the module along with the item questionnaire for scoring on the nonrelevant, somewhat relevant, relevant, and highly relevant scale. The items asked the experts to score were as follows: whether the module is suited for the intended audience’s needs, whether it effectively meets the specified learning objectives, alignment of the teaching-learning methods outlined in the proposed module with the specified competencies, the effectiveness of the suggested module in evaluating the relevant skills and appropriateness of the teaching-learning and assessment methods in the proposed module in terms of being community centred. Based on the scoring given by the experts, the Content Validity Index (CVI) was calculated, and 0.80 and above is considered excellent or relevant [9].

## Results

The Teaching-Learning module for the FAP was reviewed by the panel of external experts (10) of health professional education, and scoring was given to the item questions given to them. Experts were asked to go through the module thoroughly and give a ranking as follows: rank of one to not relevant, rank of two to somewhat relevant ranking, three to relevant and rank of four to highly relevant for each item question. Later, the investigator gave the scores as zero and one (rank one and two as score zero and rank three and four as score one), as shown in Table 2.

**Table 2:** The average proportion of items judged as relevant across the experts (n=10) CVI - Content Validity Index I-CVI - Item-Content Validity Index S-CVI/Ave - scale content validity index/Average

Items questions	Experts scoring										Experts in agreement	I-CVI
The extent to which the suggested module is well suited for the intended audience’s needs	1	1	1	1	1	1	0	1	1	1	9	0.9
The extent to which the suggested module effectively meets the specified learning objectives	1	1	1	1	1	1	1	1	1	1	10	1.0
the alignment of the teaching-learning methods outlined in the proposed module with the specified competencies	1	1	1	1	1	1	1	1	0	1	9	0.9
the effectiveness of the suggested module in evaluating the relevant skills	1	1	1	1	1	1	1	1	1	1	10	1.0
the appropriateness of the teaching-learning and assessment methods in the proposed module in terms of being community-centred	1	1	1	1	1	0	1	1	1	0	8	0.8
Total	5	5	5	5	5	4	4	5	4	4		4.6
proportion relevance	1	1	1	1	1	0.8	0.8	1	0.8	0.8	9.2	0.92
S-CVI/Ave												0.92

CVI comes in two flavours: CVI for scale (S-CVI) and CVI for the item (I-CVI). There are two ways to calculate S-CVI. The first uses the average of all I-CVI values across the scale (S-CVI/Ave). An acceptable CVI value of 0.80 and above is considered good. The CVI obtained for the given module was 0.92, considered relevant and sound [9].

## Discussion

The FAP aims to provide an experiential learning opportunity to Indian medical students towards community healthcare and to give orientation to primary healthcare. By the use of a standardized and validated teaching-learning module it is possible to implement the competency-based medical education curriculum by the NMC.

The module underwent validation with the assistance of expert panels, resulting in a calculated CVI value of 0.92, deemed highly relevant and commendable. In their study, Yalamanchili et al. [7] highlighted the perceived benefits of the FAP, noting its potential to offer valuable insights into patients’ living conditions while also inspiring students towards their future careers. Apart from various challenges, they also suggested increased faculty involvement, alongside concerns about workload and faculty requirements.

In their research to create, present, and assess a structured, validated module on communication skills for interns, Dutta et al. [10] discovered that the post-training knowledge score (16.68±2.5) was considerably more significant than the pre-training score (15.45±2.9). Chakraborty et al. [11] concluded that the FAP, by fostering compassion, cultural competence, and community engagement, equips medical students to become skilled and empathetic physicians capable of addressing the diverse healthcare needs of society. They emphasized the vital role of community medicine in implementing the FAP and acknowledged potential challenges in its execution.

Aggarwal et al. [12] conducted a study evaluating the impact of communication skills (CS) training on first-year MBBS students at a Government Medical College. Participants positively received the participant-centric, assessment-based teaching and learning module, finding it engaging and insightful. The transformative potential of an innovative Community Orientation Program (COP) as a technique of CBME was investigated by Narapureddy et al. [13]. This strategy aimed to provide medical students with early exposure to community engagement while highlighting their communication skills, observational talents, and desire to learn through group projects. The plan sought to transform conventional teaching models by motivating pupils early on and equipping them with valuable skills.

In their study on the effects of communication modules on medical students, Nayak et al. [14] concluded that the faculty felt these modules encouraged the students to develop their communication abilities. On the other hand, faculty believed that these modules might not be enough to teach communication skills. At the same time, Ramezani et al. [15] came to a similar conclusion at the end of their study regarding the effectiveness of using virtual education modules to improve communication skills instruction in medical schools. FAP would accomplish two goals: first, it would make healthcare facilities more accessible to rural communities and motivate them to seek them out; second, it would offer community-focused training to aspiring medical professionals [16].

The study’s strength is that it highlighted that the structured teaching-learning-assessment module could be relevant for training the students in a team of interprofessional teachers to meet the competencies required or needed for the family adoption program. The study’s limitations are the generalizability to all the medical institutes of the country, as the feedback from a representative sample of the entire nation has not been taken, and the implementation of the module; the effectiveness of the teaching-learning module could be affected by how well it is implemented.

## Conclusions

The Interprofessional Teaching-Learning Module is an all-encompassing collaborative approach designed to give students the fundamental information and abilities to support and assist adoptive families. The Family Adoption Program has great potential to improve patient care and move society closer to a healthier and more equitable future, in keeping with India’s goal of achieving universal health coverage if properly implemented and maintained.

## Appendices

Items/questions used to assess the module by the experts; 

Item 1 - "Please rate the extent to which the suggested module is well suited for the intended audience’s needs."

Item 2 - "Please rate the extent to which the suggested module effectively meets the specified learning objectives."

Item 3 - "Please rate the alignment of the teaching-learning methods outlined in the proposed module with the specified competencies."

Item 4 - "Please rate the effectiveness of the suggested module in evaluating the relevant skills."

Item 5 - "Please rate the appropriateness of the teaching-learning and assessment methods in the proposed module in terms of being community-centred."
